# Elevated serum levels of monocyte chemoattractant protein-1 in 71 patients 3 months after elective cardiac surgery suggest a potential link to persistent inflammation but not an increased propensity for perioperative cerebrovascular events

**DOI:** 10.3389/fmed.2025.1561886

**Published:** 2025-08-18

**Authors:** Krzysztof Laudanski, Mohamed Antar, Hossam Gad, Daniel A. Diedrich

**Affiliations:** Department of Anesthesiology and Perioperative Care, Mayo Clinic, Rochester, MN, United States

**Keywords:** cardiac surgery, monocyte chemoattractant protein 1, long-term outcome, vasculitis, neuroinflammation, neurodegeneration, stroke, cerebrovascular event

## Abstract

**Background:**

Monocyte chemoattractant protein 1 (MCP-1) plays a critical role in the transmigration of peripheral monocytes, a central mechanism underlying chronic inflammation. In this study, we investigate postoperative serum kinetics of MCP-1 as a potential contributor to postoperative neurocognitive decline, arteriosclerosis, and the development of organ failures.

**Methods:**

Seventy-one patients undergoing elective cardiac surgery were included in this study. Serum samples were collected preoperatively (t_baseline_), and postoperatively at 24 h (t_24h_), 7 days (t_7d_), and 3 months (t_3m_). MCP-1 levels were quantified in conjunction with other inflammatory markers and alarmins. Whole blood samples underwent lipopolysaccharide (LPS) stimulation to evaluate MCP-1 production capacity, and peripheral monocyte transcriptomes were analyzed. Surrogate markers of end-organ dysfunction, including markers of neurodegeneration, neuroinjury, vasculitis, and atherosclerosis, were assessed. Acute kidney failure was defined per the RIFLE criteria, and occurrences of cerebrovascular accidents (CVA), pulmonary embolism (PE), deep venous thrombosis (DVT), and dispositions were documented.

**Results:**

Cardiac surgery resulted in an acute increase in serum MCP-1 at 24 h, 7 days, and 3 months as compared to the presurgical baseline. MCP-4 levels were unchanged, while Regulated on Activation Normal T Cell Expressed and Secreted cytokine (RANTES) was significantly depleted after surgery. Except for a prior history of cerebrovascular accidents, other preoperative clinical conditions, duration of anesthesia, surgery, cross-clamp, estimated fluid loss, and transfusions did not influence t_24h_ MCP-1 serum level. Perioperative use of non-steroidal anti-inflammatory drugs and opioids affected acute serum MCP-1 levels. At 3 months, patients undergoing coronary artery bypass graft (CABG) surgery exhibited the most pronounced elevation in MCP-1 compared to other cardiac surgery. Serum IL-6 at 24 h positively correlated with MCP-1 levels measured at 24 h, 7 days, and 3 months. Additionally, markers of neurodegeneration (*τ* protein and amyloid *β*-1-40), some vascular inflammation (FGF-23 and FGF-21), and atherosclerosis (LOX-1) demonstrated correlational relationships with MCP-1. Finally, patients experiencing postoperative cerebrovascular accidents demonstrated depressed levels of MCP-1 at 24 h, 7 days, and 3 months as compared to patients recovering uneventfully.

**Conclusion:**

Serum MCP-1 levels were elevated for up to 3 months following cardiac surgery, even in patients who experienced an uneventful recovery. MCP-4 was unchanged, while RANTES depressed post-surgery. A significant correlation between MCP-1 and serum surrogate markers of brain injury, vascular inflammation, and atherosclerosis highlights MCP-1 as a potential biomarker and a possible mediator of adverse outcomes after cardiac surgery.

## 1 Introduction

Monocyte chemoattractant proteins (MCPs) regulate leukocyte behavior during acute and chronic inflammation (1–6). MCP-1 activates monocytes (MO) in the bloodstream and facilitates their translocation through blood vessels into peripheral tissues (1, 7). MCP-4 similarly recruits monocytes but typically induces chronic and atypical activation (4, 8). Another chemokine, Regulated on Activation Normal T Cell Expressed and Secreted cytokine (RANTES) potentiates leukocyte vascular transmigration (6, 9, 10). A coordinated release of chemokines promotes the migration of activated monocytes into extravascular sites, subsequently facilitating the resolution of inflammation (1, 3, 4, 6–8). Excessive chemokine levels, however, lead to over-activation and exaggerated monocyte migration into tissues. Conversely, insufficient chemokine release impairs monocyte recruitment, compromising tissue repair and healing (10, 11). Such imbalances adversely influence leukocyte composition and function, potentially affecting surgical outcomes. Ultimately, either scenario—excessive or insufficient chemokine signaling—results in inappropriate monocyte recruitment, unfavorable polarization, impaired healing, and sustained tissue inflammation (3–5, 9–14).

Imbalanced chemokine release results in unfavorable monocyte activation, adversely impacting organ function (1). Vasculitis mediated by MCP-1 compromises end-organ function through impaired nutrient delivery and increased leukocyte retention (1). Specifically, inflammation within the central nervous system (CNS) is driven by peripheral monocyte recruitment mediated predominantly by MCP-1 and MCP-4. Additionally, these chemokines promote monocyte infiltration into the subendothelial space of atherosclerotic blood vessels (4, 8, 15). Consequently, elevated MCP-1 levels correlate strongly with increased incidence of neuroinflammation, cognitive decline, transient ischemic attack (TIA), ischemic cerebrovascular accident (CVA), chronic renal failure, chronic obstructive pulmonary disease, and acute coronary syndrome (ACS). These clinical outcomes result from accelerated progression of neurodegeneration, atherosclerosis, and fibrosis, processes directly influenced by elevated MCP-1 signaling (1–3, 6–8, 13, 15–21).

MCP-1 is produced by peripheral monocytes, endothelial cells, and microglia in response to pathogens and alarmins (e.g., heat shock protein, HMGB-1, ATP), which are inevitably released during surgery due to the iatrogenic tissue damage (1, 17, 22–24). Consequently, surgical trauma triggers the production and release of chemokines, potentially compromising organ function through excessive, insufficient, or imbalanced recruitment and transmigration of inflammatory or atypical monocytes. Although theoretically, persistent elevation of MCP-1 could sustain chronic inflammation by continuously recruiting peripheral monocytes into inflamed organs, the long-term perioperative dynamics of MCP-1 remain unclear. Previous studies in cardiac surgery have demonstrated acute postoperative MCP-1 elevation; however, observation periods have typically been short (3, 23, 25, 26). Even after the initial inflammatory insult resolves, local leukocytes, and microglia may continue to produce MCP-1 due to persistent changes in gene regulatory mechanisms (16, 17). This sustained MCP-1 expression may promote ongoing neuroinflammation, vasculitis, and atherosclerosis, thus elevating the postoperative risk of cerebrovascular accidents, acute coronary syndrome, and other organ dysfunction (10, 11, 26–30). Ultimately, the severity of these complications may correlate with both the duration and magnitude of MCP-1 dysregulation (3, 30).

In this study, we hypothesized that perioperative inflammation associated with cardiac surgery would lead to elevated MCP-1 serum levels, which would gradually normalize during patient convalescence (at 7 days) as compared to other principal chemokines (2, 25, 26, 31). Additionally, we aimed to compare the dynamics of MCP-1 levels with other inflammatory markers and alarmins released during the perioperative period. We further hypothesized that postoperative MCP-1 elevations would correlate with surrogate markers of cardiac, renal, and neuronal injury and that the persistence of elevated MCP-1 levels post-surgery would be proportional to these injury markers (1, 2, 4, 6, 9, 14, 19, 23, 29). Finally, we explored whether elevated MCP-1 serum concentrations are associated with subjective cognitive complaints after cardiac surgery.

## 2 Methods

### 2.1 Studied population

This cohort study was approved by the Institutional Review Board of the University of Pennsylvania Institutional Review Board (#815686). Adult patients scheduled for elective cardiac surgery involving cardiopulmonary bypass were approached and included upon obtaining informed consent. Between April 2016 and March 2021, a total of 71 patients were enrolled. Exclusion criteria included a lack of consent, emergent procedure, history of cancer in the last 5 years, and immunocompromised status.

### 2.2 Sample collection

Blood was collected preoperatively (t_baseline_), and postoperatively at 24 h (t_24hr_), 7 days (t_7d_), and at 3 months (t_3m_). Blood samples were collected using the Vacutainer™ system prefilled with heparin anticoagulant (BD; Franklin Lakes, NJ) and stored at 4°C until processed within 4 h of collection.

### 2.3 Clinical and outcome data collection

Demographic, clinical, surgical, anesthetic, and perioperative data were collected from the electronic health record (EHR). Morphine equivalents were calculated from opioid medications administered within the first 24 h post-surgery. The APACHE II score was determined upon ICU admission, as well as 24 and 48 h postoperatively (32). Cerebrovascular events (CVA) diagnosed pre- and postoperatively were documented from EHR data. Mortality was assessed at 28 days and at 3 months following surgery. Acute kidney injury (AKI) was defined according to the RIFLE criteria (33). At the 3-month follow-up, patients were asked about subjective changes in their cognitive functioning, memory, and sleep quality compared to their preoperative baseline (defined as worse, same, *vs* improved).

### 2.4 Assessment of biological variables

Serum levels of MCP-1, MCP-4, and RANTES were quantified using an ELISA kit (Biolegend, San Diego, CA). Monocytes (Mos) were separated from other peripheral blood cell types using a Ficoll–Hypaque density gradient (34). MOs were further purified by removal of B cells, NK cells, and T cells using the negative separation technique to reduce selection bias as described previously (34). MCP-1 mRNA was isolated for transcriptomic analysis via RNA-seq performed commercially (Novogene, Davis, CA) as part of a larger project. mRNA was isolated using a commercial kit (Zymo Research, Irvine, CA) after Triazol preservation. The nucleic material’s quality was measured by 260/280 absorption. First-strand cDNA was synthesized using a random hexamer primer and M-MuLV Reverse Transcriptase (RNase H), followed by second-strand synthesis with DNA Polymerase I and RNase H, and purification with AMPure XP beads. Double-stranded cDNA was converted to blunt ends, adenylated at the 3′ end, and ligated with NEBNext Adaptor. Library fragments were purified with the AMPure XP system and obtained by PCR amplification. For strand-specific libraries, dTTP was replaced by dUTP during second-strand synthesis. After converting cDNA to blunt ends and adenylating, NEBNext Adaptor was ligated, and the second strand cDNA was digested with USER enzyme. After constructing the library, dilute it to 1.5 ng/ul using Qubit2.0 results and check the insert size with Agilent 2,100. Q-PCR is used to ensure the library’s effective concentration is above 2 nM for quality assurance. If it meets standards, load it into a sequencer (Illumina, San Diego, CA).

Alarmins indicating tissue damage, specifically Hsp-70 (Life Technologies, Waltham, MA) and HMGB-1 (Aviva Technologies, San Diego, CA), were measured using ELISA (29). Non-specific inflammatory responses were through IL-6 and C-reactive protein. Surrogate markers of end-organ dysfunction—including FGF-21, FGF-23, VEGF, *τ*, p181τ, UC-HL, GFAP, TDP-43, and NT-BNP were analyzed using multiplex technology (ThermoFisher, Waltham, MA) on MagPix device (Luminex, Northbrook, IL) as the surrogates of end-organ failure (30).

### 2.5 WBS production of MCP-1 in response to pathogen stimulation

Whole blood samples (500 μL) were stimulated with lipopolysaccharide (LPS; 50 ng/mL; Lonza, Wayne PA), HMGB-1 (R&D, Minneapolis, MN), or H3N2 [0.5mcg/ml] (BEI Resources, Bethesda, MD) or left unstimulated for 18 h in the orbital shaker at 37°C and 5% CO_2_ (35). Following stimulation, blood was centrifuged at 2000x*g* for 5 min, and plasma was collected. IL-6 and TNFα levels were analyzed using a multiplex platform (Luminex, Northbrook, IL) while MCP-1 using ELISA (Biolegend, San Diego, CA).

### 2.6 Statistical analysis

Shapiro–Wilk W and K-S tests assessed the variables’ parametric characteristics. Variables are expressed as mean±SD and compared using *t*-Student (*t*[*n*]) or ANOVA or as median (M_e_) and interquartile ranges (IR) with the U-Mann–Whitney test (U[n1; n2]) or Wilcoxon Signed-Rank Test used for non-parametric data. In general, non-parametric tests were used predominantly based on data characteristics to increase the robustness of the findings. *d*-Cohen was used to assess the degree of difference (36, 37). ANOVA was utilized for multiple variables, with a statistic of *η*^2^ determining the significance of the association. Since our primary hypothesis was the normalization of MCP-1 to presurgical baseline, we utilized longitudinal design in the statistical approach. The correlation coefficients were used to assess relationships. *p*-value less than 0.05 was considered statistically significant for two-tailed hypotheses unless a specific one-tailed hypothesis was formulated (37). Considering that our hypotheses assume the return of the MCP-1 levels to baseline, traditional power analysis is not applicable (36, 37). Prior studies enrolled fewer individuals than our study (12, 14, 23, 25–27, 31, 34, 35). Furthermore, in our statistical approach, we were conservative and could not account for several perioperative covariables, though longitudinal design minimizes heterogeneity related to preoperative conditions (37).

## 3 Results

### 3.1 Characteristics of the studied cohort

71 individuals were enrolled in the study. Table 1 presents their essential demographic and clinical characteristics.

**Table 1:** Demographical and clinical characteristic of studied population.

Demographics (*N* = 71)		
Age (Mean±SD) [Years]		63.4 ± 12.9
Over 60 Years [%]		67.6%
Sex	Male [%]	80.3%
	Female [%]	19.7%
Race	White [%]	85.9%
	African American [%]	4.2%
	Other / Asian / Unknown [%]	9.8%
Preexisting conditions		
Weight (Mean±SD) [kg]		85.12 ± 19.68
BMI (Mean±SD)		27.9 ± 4.9
Charlson Comorbidity Index (Mean±SD)		3.96 ± 2.1
ACS/MI [%]		16.9%
CHF [%]		19.7%
PVD [%]		9.9%
CVA/TIA [%]		12.7%
COPD [%]		5.7%
DM [%]		25.4%
Anesthesia & surgery data		
Duration of anesthesia (Mean±SD) [min]		352.59 ± 101.89
Duration of surgery (Mean±SD) [min]		2249.12 ± 93.83
Duration of cardiopulmonary bypass (Mean±SD) [min]		134.4 ± 61.4
Coronary artery bypass surgery [*n*]		33
Mitral valvuloplasty & replacement [*n*]		11
Aortic valvuloplasty & replacement [*n*]		24
Estimated Blood Loss (Mean±SD) [mL]		214.221 ± 337
Perioperative management		
Transfusions during surgery		
Packed Red Blood Cells (Mean, IQR) [mL]		149.17, 0
Fresh Frozen Plasma (Mean, IQR) [mL]		40.44, 0
Total crystalloid during surgery (Mean, IQR) [mL]		1271.67, 600
Clinical Care during 24 h post-surgery		
Packed Red Blood Cells (Mean, IQR) [mL]		15, 0
Fresh Frozen Plasma (Mean, IQR) [mL]		0, 0
Corticosteroid Administration [% of all cases]		10%
Ketorolac Administration [% of all cases]		10%
Acetaminophen Administration [% of all cases]		78.6%
Acetylsalicylic acid Administration [% of all cases]		72.9%
Opioids Administration (Mean±SD) [mg]		683.86 ± 259.2
Benzodiazepine administration (Mean±SD) [mg]		0.53 ± 2.79
Outcome at 28 days		
LOS ICU (Mean±SD) [Days]		5.34 ± 22.9
LOS Hospital (Mean±SD) [Days]		10.59 ± 24.67
Discharged home/In the healthcare facility/Readmission [%]		94.4% / 4.2% / 1.4%
Outcome at 3 months		
Discharged home/In the healthcare facility/Readmission [%]		94.3%/ 2.9%/ 2.9%

### 3.2 The effect of presurgical conditions on baseline MCP-1

The preoperative level of serum MCP-1 was not affected by age, gender, race, or BMI.

**Figure 1:**
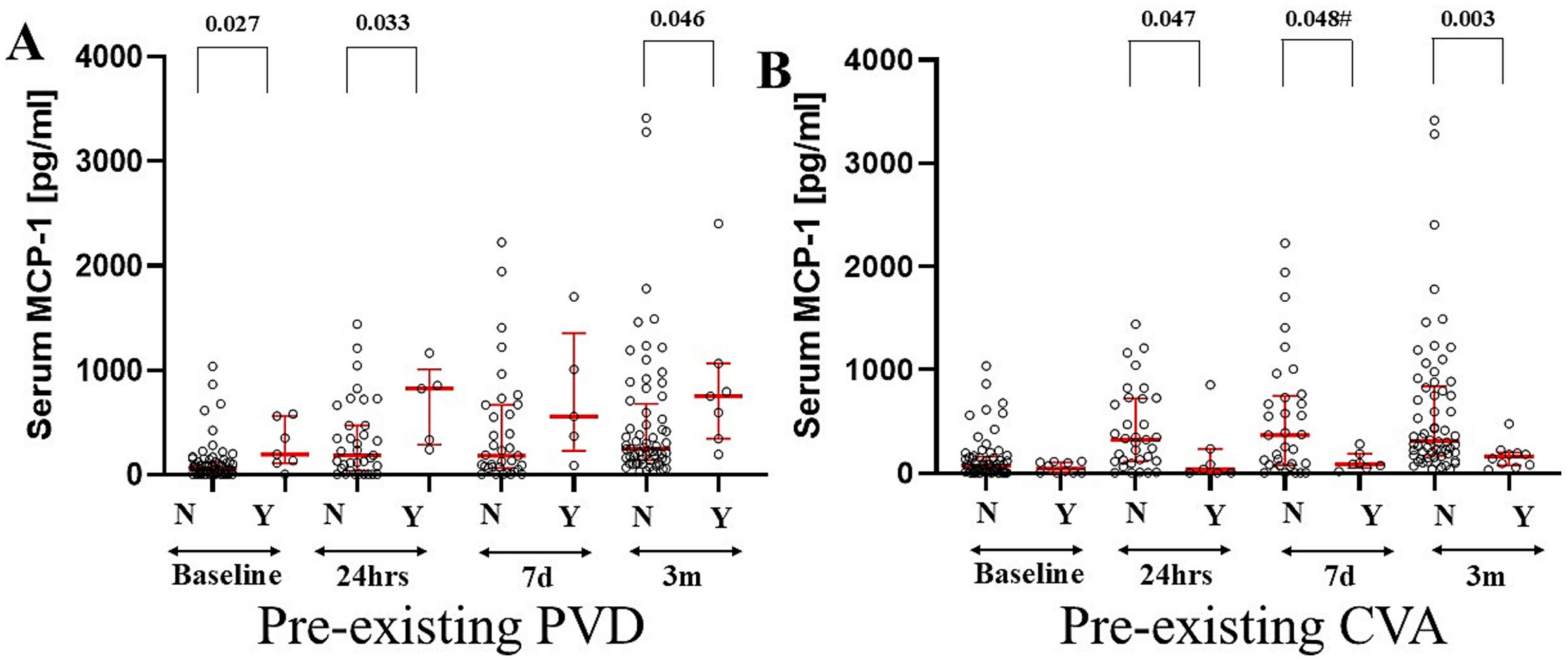
Preexisting peripheral vascular disease (PVD) resulted in an increased level of serum MCP-1 at all studied time points except t_7d_ as compared to patients without pre-diagnosis of PVD **(A)**. Patients with a pre-existing history of cerebrovascular accident (CVA) demonstrated diminished levels of serum MCP-1 at all post-operative time points **(B)**. The red bars represent the median and upper and lower quartiles of the individual’s values. Statistically significant differences between groups at each sample point are displayed using bars and exact *p* values. # signifies that one-sided *p*-value was calculated.

Serum MCP-1 levels were significantly higher in patients without peripheral vascular disease (PVD) at baseline MCP-1_No-PVD_ *vs*. MCP-1_PVD_ (U[64; 7] = 336; *p* = 0.03), 24 h MCP-1_No-PVD_ *vs*. MCP-1_PVD_ (U[35; 5] = 139; *p* = 0.035) (Figure 1A). This difference remained borderline significant at 3 months MCP-1_No-PVD_ *vs*. MCP- _PVD_ (U[64; 7] = 327; *p* = 0.047). Additionally, patients with a history of cerebrovascular events had depressed levels of MCP-1 at 24 h (MCP-1_No-CVA_ = 419.1 ± 393.9 *vs* MCP-1_CVA_ = 175.39 ± 310.58; U[33; 7] = −1.9*; p =* 0.048) and 3 months (MCP-1_No-CVA_ = 572 ± 565.34 *vs* MCP-1_CVA_ = 124.03 ± 67.26; U[61; 10] = −2.8*; p =* 0.004) compared to patients without prior cerebrovascular accidents history (Figure 1B).

### 3.3 Changes of serum MCP-1 after surgery or in response to stimulation

**Figure 2:**
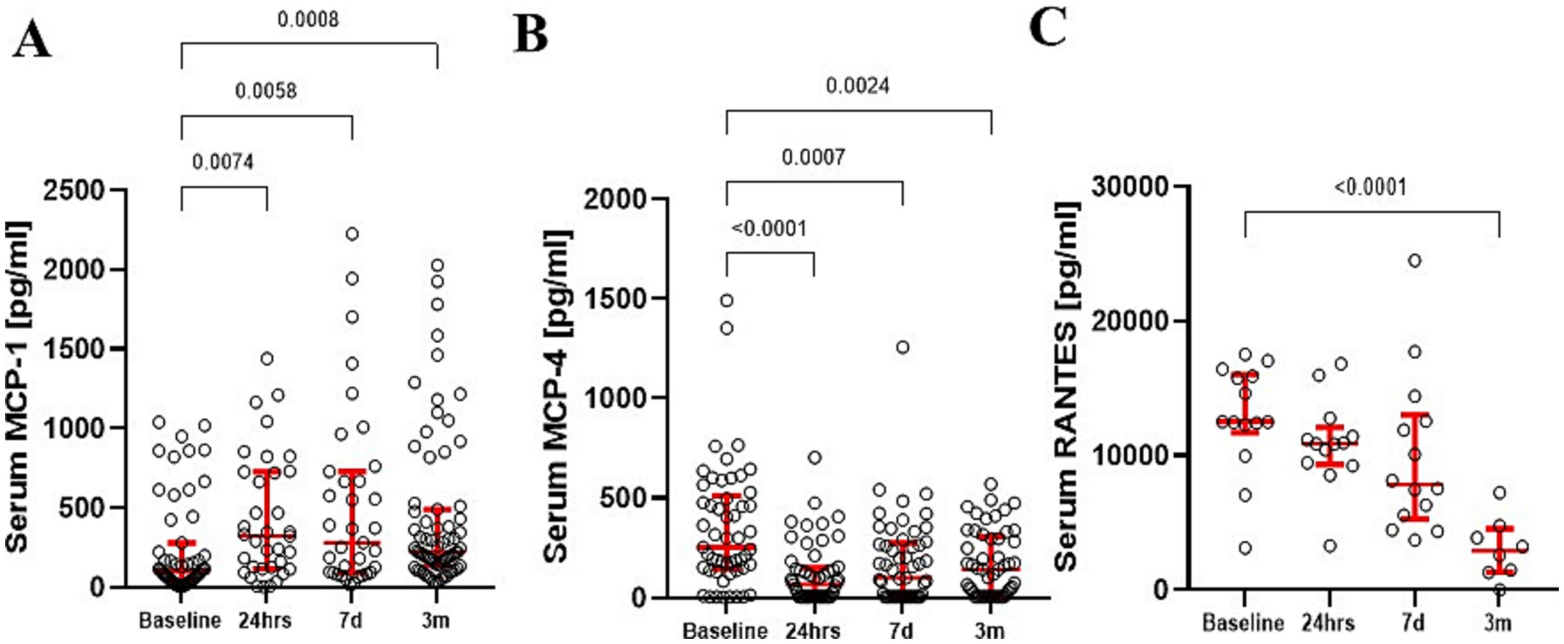
Serum MCP-1 levels demonstrated a significant increase up to 3 months after surgery **(A)**. In contrast, serum MCP-4 **(B)** and RANTES **(C)** levels significantly dropped over time. The red bars represent the median and upper and lower quartiles of the individuals’ values. Statistically significant differences between values before surgery and post-procedure sampling points are displayed using bars and exact *p* values below 0.05.

Serum level of MCP-1 significantly increased following surgery across sample times [KW (3; 222) = 43.93; *p <* 0.001] (Figure 2A). Pairwise comparisons revealed significant MCP-1 elevations 24 h (*p* = 0.001), 7 days (*p* = 0.001), and at 3 months (*p* = 0.001). *d*-Cohen analysis demonstrated the most profound impact at 24 h (*d* = 0.793), followed by 7 days (*d* = 0.68), and still significant at 3 months (*d* = 0.641) postoperatively when compared to preoperative baseline levels. Additionally, MCP-1 mRNA expression in peripheral blood monocytes increased postoperatively but returned to baseline by 3 months. In contrast, serum MCP-4 (Figure 2B) showed no changes over time, while RANTES demonstrated a decrease at 3 months as compared to the baseline (Figure 2C).

In multivariate analyses (adjusted for age, anesthesia duration, cardiopulmonary bypass duration, aortic cross-clamp duration, cardiac arrest occurrence, and perioperative medication usage), none of these factors significantly influenced MCP-1 serum levels at any time points (Supplementary material 1). Interestingly, serum MCP-1 concentrations at 24 h and 7 days negatively correlated with APACHE scores at ICU admission (t_0h_: *r* = −0.422; *p* = 0.008, t_24h_: *r* = −0.517; *p* = 0.001). Additionally, APACHE scores at 24 h correlated negatively with MCP-1 levels at 7 days (*r* = −0.471; *p* = 0.03), suggesting patients with higher clinical severity had lower MCP-1 elevations.

**Figure 3:**
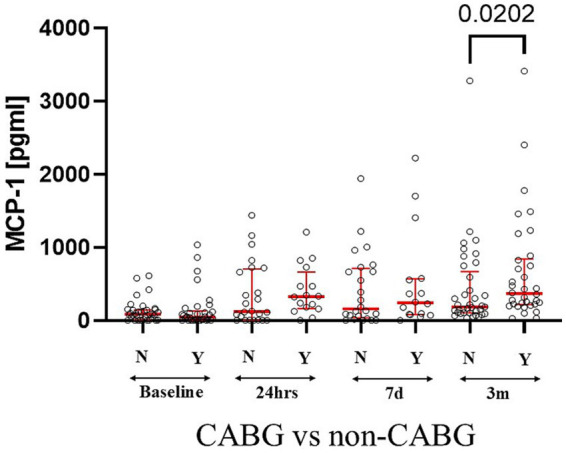
CABG patients had increased levels of serum MCP-1 as compared to other types of cardiac surgeries at 3 months. Statistically significant differences between groups at each sample point are displayed using bars and exact *p* values. # signifies that one-sided *p*-value was calculated.

Perioperative intake of benzodiazepines, morphine, acetaminophen, and ketorolac did not correlate with MCP-1 levels at any time. Serum MCP-1 levels at 3 months showed a significant increase in patients with CABG vs. other cardiac surgery patients (MCP-1_CABG_ *vs*. MCP-1_No-CABG_ U[37; 34] = 830; *p* = 0.02) (Figure 3). MCP-1 levels at 7 days are increased in patients taking nonsteroidal anti-inflammatory drugs versus patients not taking them (M_e_ MCP-1_NSAID_ = 28 *vs* MCP-1_No-NSAID_ = 12; U[28; 12] = 2.245; *p* = 0.025) and 3 months (MCP-1_NSAIDS_ = 51*vs* MCP-1_No-NSAID_ = 19 U[51; 19] = 2.635; *p* = 0.008).

**Figure 4:**
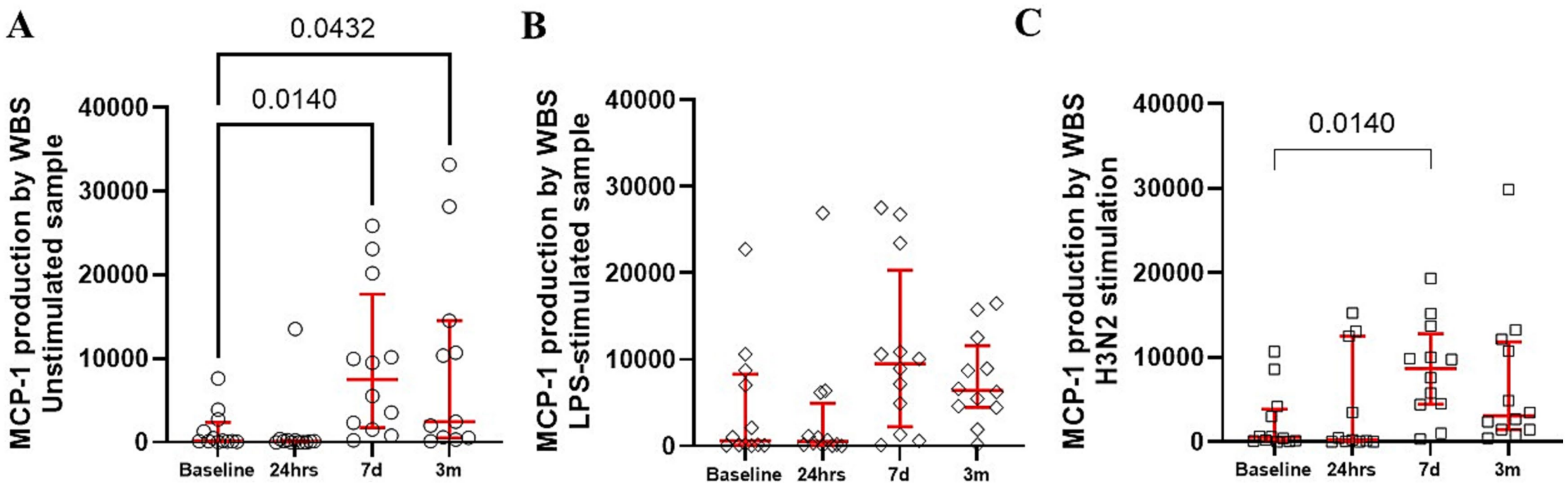
Production of MCP-1 by whole blood was constitutively upregulated **(A)** with only modest changes in response to bacterial **(B)** and viral stimuli **(C)**. The red bars represent the median and upper and lower quartiles of the individuals’ values. Statistically significant differences between values before surgery and post-procedure sampling points are displayed using bars and exact *p* values below 0.05.

Production of MCP-1 in unstimulated blood samples were elevated (Figure 4A). However, stimulation of whole blood samples with LPS or H3N2 resulted in variable MCP-1 production at the different postoperative times, without a consistent pattern (Figures 4B,C).

### 3.4 Correlations of MCP-1 and perioperative inflammation

**Figure 5:**
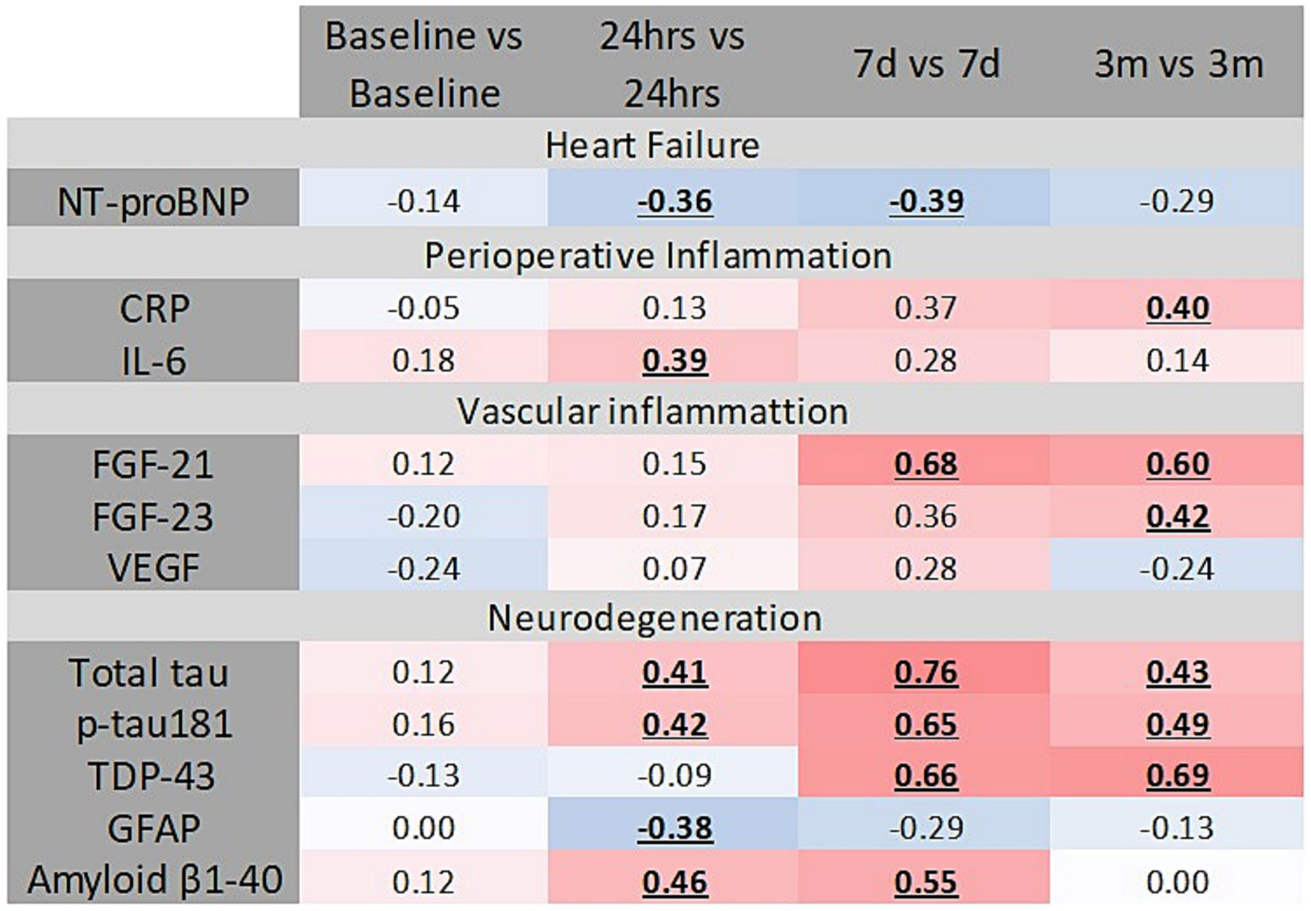
Several correlations were detected between inflammation, vascular inflammation, heart failure, and neurodegeneration markers and serum MCP-1. Statistically significant correlations are bolded and underlined. Intensity of color on the blue-to-red spectrum reflects the degree of association.

We observed no correlation between serum levels of tissue damage markers HMGB-1, Hsp-70, and MCP-1 at 24 h (data not shown). However, IL-6 levels measured at 24 h postoperatively demonstrated a positive correlation with MCP-1 levels at 24 h (*r* = 0.39; *p* = 0.019), 7 days (*r* = 0.43; *p* = 0.009), and 3 months (*r* = 0.31; *p* = 0.02) (Figure 5). No significant correlation was detected between the serum level of CRP at 24 at any time point.

### 3.5 Changes of MCP-1 with surrogates of heart failure, brain injury, brain degeneration, and macrophage characteristics

Several markers of vascular inflammation demonstrated positive correlations with MCP-1 serum levels; specifically, FGF-23 and FGF-21 correlated positively at multiple time points. In contrast, VEGF exhibited a negative correlation with MCP-1 at 24 h but a positive correlation at 3 months (Figure 5). No correlation was found between MCP-1 and serum levels of UC (data not shown).

Baseline levels of MCP-1 positively correlated with preoperative serum levels of total *τ*, phosphorylated τ protein (at 181 domain), Amyloid β1-40, GFAP, and TDP-43 (Figure 5). At 24 h post-surgery, serum total τ protein significantly correlated with serum MCP-1 (Figure 5). Additional correlation occurred at 7 days (*r* = 0.43; *p* = 0.011), and 3 months (*r* = 0.35; *p* = 0.014). Serum MCP-1 levels at 24 h correlated with p181τ at 24 h (Figure 5). Amyloid β1-40 levels at 3 months correlated positively with MCP-1 levels at all postoperative time points (Figure 5). TDP-43 levels at 3 months correlated with MCP-1 levels at 24 h, 7 days (*r* = 0.61; *p* = 0.001), and 3 months (*r* = 0.69; *p* = 0.001).

### 3.6 Clinical outcomes and disposition

There was no correlation between MCP-1 levels at any point and postoperative AKI or elevated NT-BNP (data not shown).

Patients reporting subjective postoperative decline exhibited lower serum MCP-1 at t_24hr_ (M_e_ MCP-1_24h_ = 82.6) compared to patients without perceived cognitive deterioration (M_e_ MCP-1_24h_ = 102), resulting in a statistically borderline difference (U[44; 6] = 1.72; *p* = 0.043 one-sided). However, subjective perceptions of sleep quality, memory, and overall recovery showed no association with MCP-1 serum levels at any sampling time point (data not shown).

**Figure 6:**
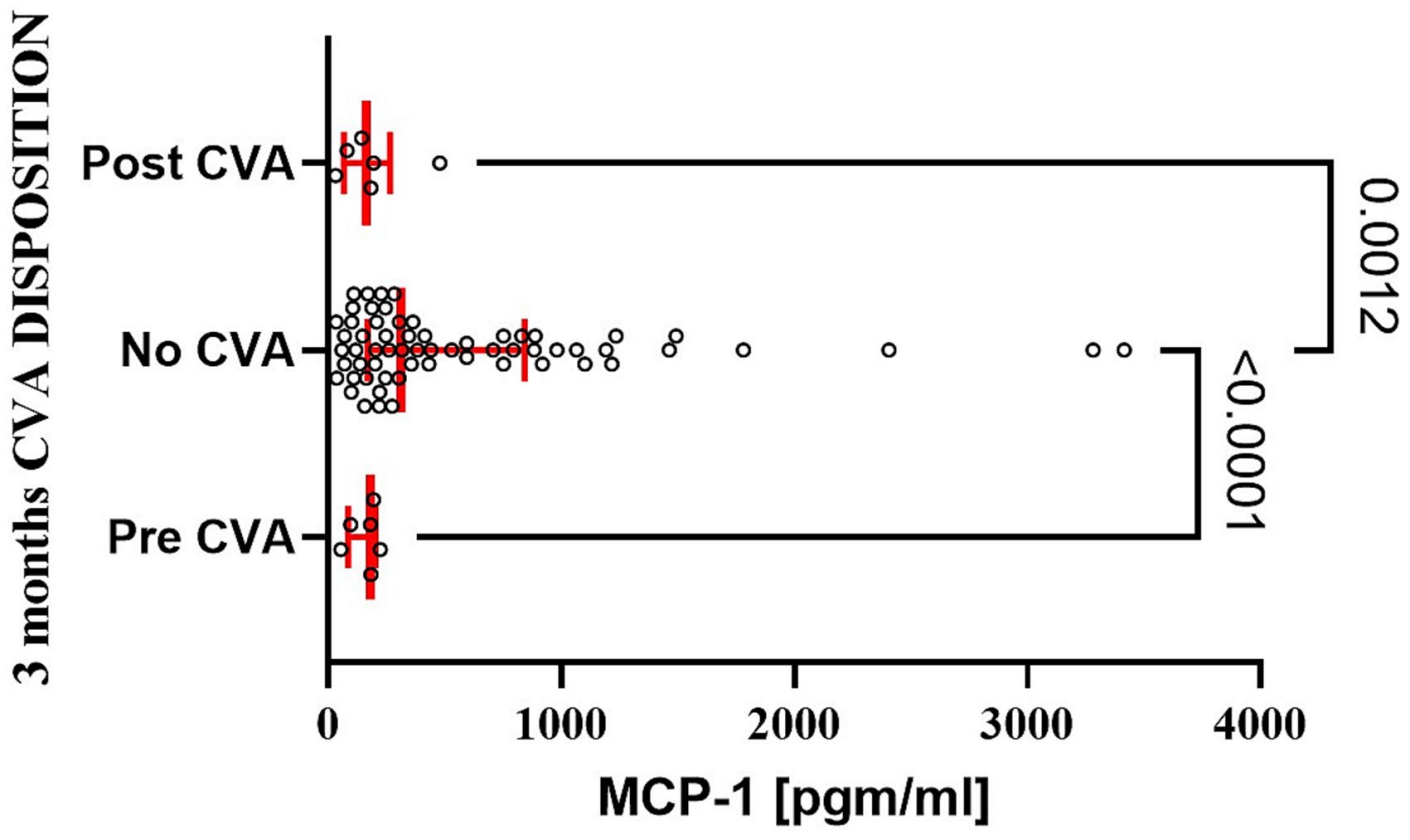
Patients experiencing postoperative stroke had a significant decline in serum MCP-1 level at 3 months as compared to patients with uneventful recovery. The bar represents the median, while the whiskers represent the interquartile ranges. Statistically significant differences between groups at each sample point are displayed using bars and exact *p* values. # signifies that one-sided *p*-value was calculated.

A limited number of patients experienced postoperative complications at 28 days and 3 months. MCP-1 serum levels did not differ between patients discharged home uneventfully and those experiencing complications, including cerebrovascular events, hospital readmission, or death (data not shown). Interestingly, patients experiencing cerebrovascular events within the first three postoperative months demonstrated significantly lower serum MCP-1 levels compared to patients without such events (Figure 6). However, there were no significant differences in MCP-1 levels when comparing patients with postoperative cerebrovascular accidents to those with a history of preoperative cerebrovascular accidents.

## 4 Discussion

Our work demonstrated, for the first time, that post-operative changes in serum MCP-1 levels can persist for over 3 months, extending well beyond the traditional 28-day mortality window and into the recovery period. Prior research reported acute elevations in serum MCP-1 levels that diminished within 24 h (2, 14, 26, 28, 31). Interestingly, our study found a sustained MCP-1 increase that was especially pronounced in patients undergoing coronary artery bypass grafting (CABG), suggesting procedure-specific MCP-1 dynamics (26, 28). In contrast, serum MCP-4 levels remained unchanged, while RANTES levels were notably low. The reasons underlying this prolonged MCP-1 elevation remain unclear, though it might reflect differences in tissue injury, the diverse role of studied chemokines, or inflammation intensity between coronary artery procedures and other cardiac surgeries (6–8, 11, 18, 26, 28). However, the exact mechanism driving these differences remains speculative and warrants further investigation. While this observation may be incidental, vascular surgeries are associated with more robust MCP-1 production (28). On the other hand, more significant interventions in cardiac tissue, as seen in CABG, may contribute to this increased release of MCP-1 by exaggerated tissue damage. An observed negative correlation with alarmin does not support such an explanation (23). The production of MCP-1 appears constitutional, as bacterial or viral stimuli produced inconsistent responses, suggesting a baseline intrinsic regulation rather than pathogen-dependent induction, at least in MO. However, this finding aligns with the complexity of chemokine regulation, suggesting that MCP-1 elevation might reflect compensatory or rebound inflammatory processes. The concomitant lack of changes in MCP-4 and depressed RANTES indicates that postoperative chemokines may alter their compositions, with singular chemokine levels not being very consequential.

Alternatively, iatrogenic influences could impact MCP-1 over time in the long term (13, 17, 28). The persistence of MCP-1 serum levels may also be related to changes in promoter regulation, retention of alarmin, or smoldering inflammation (3, 22, 23, 38). Interestingly, we observed elevated MCP-1 levels among patients who took NSAIDs perioperatively despite the expectation that they would suppress inflammation and, consequently, MCP-1 production (39). This finding aligns with the complexity of chemokine regulation, suggesting that MCP-1 elevation might reflect compensatory or rebound inflammatory processes. Opioids, another commonly used postoperative medication, would theoretically suppress MCP-1 production, although this effect was not explicitly observed in our analysis (39). Finally, persistent postoperative inflammation, as evidenced by correlations between MCP-1 and IL-6, could represent the dominant factor driving prolonged MCP-1 elevation in cardiac surgery patients (3).

Previous studies have linked elevated MCP-1 levels with delirium, acute kidney injury, cardiac dysfunction, and increased mortality (1–6, 8, 27). In our study, we did not observe a correlation between MCP-1 levels and these clinical outcomes. Associations with surrogates of vascular inflammation were variable and inconsistent. Importantly, we found significant correlations between MCP-1 levels and serum surrogate markers of neurodegeneration across multiple postoperative time points, consistent with prior reports highlighting MCP-1’s involvement in perioperative central nervous system dysfunction. Interestingly, patients exhibiting lower postoperative MCP-1 serum levels reported more pronounced subjective cognitive decline. This unexpected finding might suggest alternative mechanisms or reflect individual susceptibility to postoperative neurocognitive changes (3). Further investigation into MCP-1 dynamics and formal cognitive assessments could clarify these relationships. Formal testing of postoperative cognitive performance can be particularly needed.

Patients who experienced cerebrovascular events demonstrated lower serum MCP-1 at both 24 h and 3 months postoperatively. Previous literature has primarily associated elevated, not suppressed, MCP-1 levels with increased cerebrovascular events, making our findings of decreased MCP-1 in cerebrovascular accident patients unexpected (1–3, 7, 28). Our analysis included relatively few subjects with postoperative cerebrovascular events, limiting the generalizability of this observation. We were also limited in the analysis of data documented in electronic medical records. Consequently, it was impossible to establish more details regarding the prior incidence of CVA. Previous literature linking MCP-1 to CVSs has relied primarily on indirect evidence or short-term postoperative observations, underscoring the necessity for validation in larger patient cohorts with extended observational periods and follow-up assessments (13, 15, 16, 19). How reduced MCP-1 levels relate to the incidence of cerebrovascular accidents remains unclear.

Given that MCP-1’s pleiotropic functions, this observed suppression may reflect impaired immune activation, potentially resulting in suboptimal monocyte recruitment critical for tissue repair and regeneration in the postoperative phase (1, 11, 13). Due to the longitudinal nature of our study, we were able to demonstrate that prior cerebrovascular events correlated with persistently depressed MCP-1 levels, although baseline differences were not explicitly controlled (1, 2, 17, 26). While MCP-1 is generally regarded as harmful due to its association with vascular inflammation, atherosclerosis, and neurodegeneration, it also plays essential roles in physiological processes such as inflammation resolution and cardiac remodeling after surgery (11, 13, 15). Thus, our findings highlight the complexity of MCP-1’s role, suggesting that CVA-related MCP-1 suppression might represent a deficit rather than a protective effect (3, 6, 13–16, 20, 27). Our results warrant further investigation to understand the nuanced clinical implications of MCP-1 regulation and its interplay with immune system performance during the perioperative period (1, 3).

The mechanisms underlying postoperative suppression of serum MCP-1 need to be established. MCP-1 expression is predominantly regulated by NF-κB activation, a transcriptional pathway that is also known for the production of other cytokines such as IL-6 and TNFα. Although peripheral monocytes stimulated under resting conditions demonstrated increased MCP-1 production, we did not observe corresponding elevations in IL-6 and TNFα. We also noted the effect of NSAIDS, but not opioids, on perioperative MCP-1 dynamics (39). This suggests alternative regulatory mechanisms beyond NF-κB signaling alone, such as promoter methylation, histone modifications, or miRNA-mediated processes (1, 17, 38).

Our study has several limitations. First, the relatively small sample size limited our ability to perform robust multivariate analysis. Future studies should explicitly factor in preexisting CVAa as an essential variable in a larger cohort. Another limitation was the lack of control over the use and dosage of antifibrinolytics, transfusions, and fluid resuscitation, which may have influenced serum MCP-1 levels (4, 26, 39). The sample study is too small to account for several perioperative variables in a definitive way. In the case of several medications, we are unable to verify compliance despite their effect on MCP-1 production (39, 40). Some of the statistical subgroups have significantly different numbers of individuals. Though we relied heavily on non-parametric tests to show the statistical significance of observed differences, a more extensive study is needed to establish definitive associations between MCP-1 levels and clinical complications (37). Due to the data heterogeneity, we refrained from engaging in several other techniques accounting for covariables. Although elevated MCP-1 levels positively correlated with some surrogate markers of end-organ injury, our study did not definitively establish a causal link between elevated MCP-1 and clinical outcomes, partly due to the low incidence of perioperative complications. Also, utilizing sophisticated cognitive testing, visualization of atherosclerosis progress, and microscopic examination looking for potential vasculitis will be critical in establishing the clinical relevance of our findings (30). Utilizing biomarkers surrogates of end-organ dysfunction is acceptable but only at the early stages of research investigation. Finally, the cellular origin of postoperative MCP-1 elevation remains uncertain, and warrants further investigation, as monocytes, endothelial cells, and microglia are all possible sources.

Despite these limitations, our study has notable strengths. The longitudinal design enabled the identification of prolonged MCP-1 elevation up to 3 months postoperatively, which significantly reduced confounding data. Assessing MCP-1 at both the protein and RNA levels provided robust support for our findings. Furthermore, our results aligned with prior studies that demonstrated an acute postoperative increase in MCP-1 (1, 11). We also validated prior studies showing link between vascular manipulation and elevation in MCP-1 (26, 28). Finally, the significant elevation of MCP-1 observed among patients without peripheral vascular disease further underscores MCP-1’s potential role in vascular pathology. Future studies with larger cohorts, controlled perioperative medications, and rigorous multivariate analyses, including the impact of preexisting cerebrovascular accidents, are warranted to clarify MCP-1’s role in postoperative recovery and complications.

## 5 Conclusion

In summary, MCP-1 levels increased following cardiac surgery and remained elevated for up to 3 months, even in patients with uneven recoveries. This sustained elevation correlated with surrogate markers of neurodegeneration and vascular inflammation, suggesting MCP-1 as a potential mediator or biomarker of postoperative organ dysfunction. Notably, a history of cerebrovascular accidents emerged as a significant confounding factor affecting MCP-1 dynamics. Future studies are necessary to elucidate the underlying mechanisms, clarify the impact of preoperative cerebrovascular accidents on MCP-1 regulation, and determine the clinical implications of protracted changes in postoperative chemokine composition (MCP-1↓↓, MCP-4↓↑, RANTES↑↑) on postoperative outcomes.

## Data Availability

The raw data supporting the conclusions of this article will be made available by the authors, without undue reservation.

## Glossary

ACS: Acute Coronary Syndrome
BMI: Body Mass Index
CABG: Coronary Artery Bypass Graft
CNS: Central Nervous System
CRP: C-Reactive Protein
CVA: Cerebrovascular Accident
DVT: Deep Vein Thrombosis
*d*-Cohen: Cohen’s *d* statistics
TDP-43: TAR DNA-Binding Protein 43
WBS: Whole Blood Stimulation
FGF: Fibroblast Growth Factor
GFAP: Glial Fibrillary Acidic Protein
HMGB-1: High-Mobility Group Box
Hsp-70: Heat Shock Protein 70
IL-6: Interleukin 6
IR: Interquartile Range
KW: Kruskal-Wallis Test
LOX-1: Lectin-Like Oxidized Low-Density Lipoprotein Receptor-1
LPS: Lipopolysaccharide
Me: Median
MCP-4: Monocyte Chemoattractant Protein 4
MO: Monocytes
NCAM-1: Neural Cell Adhesion Molecule 1
NT-BNP: N-Terminal Pro-B-Type Natriuretic Peptide
NSAIDS: Nonsteroidal Anti-Inflammatory Drugs
PE: Pulmonary Embolism
PVD: Peripheral Vascular Disease
RANTES: Regulated on Activation, Normal T Cell Expressed and Secreted
RIFLE: Risk, Injury, Failure, Loss, End-Stage Renal Disease
RNA-seq: RNA Sequencing
SD: Standard Deviation
TIA: Transient Ischemic Attack
Th2: Helper Cell Type 2
TNFα: Tumor Necrosis Factor Alpha
τ: Tau Protein
p181τ: Tau Protein Phosphorylated at Thr181
UC-HL: Ubiquitin C-terminal Hydrolase L1
VEGF: Vascular Endothelial Growth Factor
ELISA: Enzyme-Linked Immunosorbent Assay
