# Supplementary material for: Elevated serum levels of monocyte chemoattractant protein-1 in 71 patients 3 months after elective cardiac surgery suggest a potential link to persistent inflammation but not an increased propensity for perioperative cerebrovascular events
Source: Front Med (Lausanne). 2025 Aug 18;12:1561886. doi: 10.3389/fmed.2025.1561886 (PMC12402827; doi:10.3389/fmed.2025.1561886)
Supplement: Supplementary file 1 [file Data_Sheet_1.docx]

Supplementary Material

# Supplementary Data

## Supplementary Figures


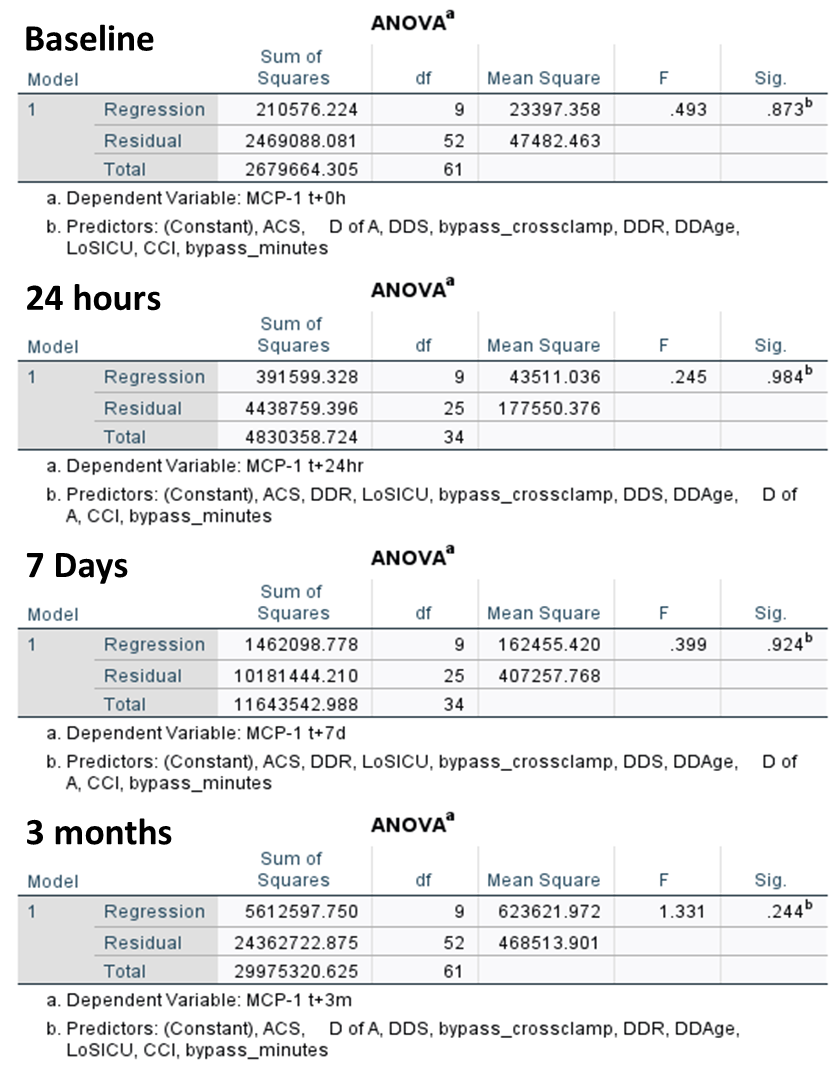


**Supplementary Figure 1.** Regression analysis of age, sex, race, Charlson Comorbidity Index, length of stay in the ICU, and duration of anesthesia, cardio-pulmonary bypass, cross-clamp against MCP-1 levels.
